# Remodeling of Mitochondrial Plasticity: The Key Switch from NAFLD/NASH to HCC

**DOI:** 10.3390/ijms22084173

**Published:** 2021-04-17

**Authors:** Miriam Longo, Erika Paolini, Marica Meroni, Paola Dongiovanni

**Affiliations:** 1 General Medicine and Metabolic Diseases, Fondazione IRCCS Ca’ Granda Ospedale Maggiore Policlinico, Pad. Granelli, Via F Sforza 35, 20122 Milan, Italy; longo.miriam92@gmail.com (M.L.); erika.paolini@unimi.it (E.P.); maricameroni11@gmail.com (M.M.); 2 Department of Clinical Sciences and Community Health, Università degli Studi di Milano, Via Francesco Sforza 35, 20122 Milano, Italy; 3 Department of Pharmacological and Biomolecular Sciences, Università degli Studi di Milano, Via Balzaretti 9, 20133 Milano, Italy

**Keywords:** NAFLD, NASH, HCC, mitochondrial dynamics, hepatocytes, KCs, HSCs, apoptosis, metabolic reprogramming, Warburg effect

## Abstract

Hepatocellular carcinoma (HCC) is the most common primary malignancy of the liver and the third-leading cause of cancer-related mortality. Currently, the global burden of nonalcoholic fatty liver disease (NAFLD) has dramatically overcome both viral and alcohol hepatitis, thus becoming the main cause of HCC incidence. NAFLD pathogenesis is severely influenced by lifestyle and genetic predisposition. Mitochondria are highly dynamic organelles that may adapt in response to environment, genetics and epigenetics in the liver (“mitochondrial plasticity”). Mounting evidence highlights that mitochondrial dysfunction due to loss of mitochondrial flexibility may arise before overt NAFLD, and from the early stages of liver injury. Mitochondrial failure promotes not only hepatocellular damage, but also release signals (mito-DAMPs), which trigger inflammation and fibrosis, generating an adverse microenvironment in which several hepatocytes select anti-apoptotic programs and mutations that may allow survival and proliferation. Furthermore, one of the key events in malignant hepatocytes is represented by the remodeling of glucidic–lipidic metabolism combined with the reprogramming of mitochondrial functions, optimized to deal with energy demand. In sum, this review will discuss how mitochondrial defects may be translated into causative explanations of NAFLD-driven HCC, emphasizing future directions for research and for the development of potential preventive or curative strategies.

## 1. Introduction

Hepatocellular carcinoma (HCC) is the main subtype of liver tumor and the third-leading cause of cancer death worldwide, whose incidence reflects the etiologies of liver diseases and their geographical distribution [1,2]. The Asian population has the highest HCC prevalence, and it is amenable to viral hepatitis in more than 50% of cases. Viral hepatitis C (HCV) and alcohol abuse prevail in western countries, where HCC is much less frequent [2]. However, the global spreading of obesity and metabolic syndrome (MetS) have rapidly increased the incidence of nonalcoholic fatty liver disease (NAFLD) over the past two decades and, in parallel, that of NAFLD-related HCC in industrialized society due also to the development of anti-viral therapies and effective HBV immunization programs. Nowadays, NAFLD is the most common chronic liver disorder, affecting 25–30% of te general population, and it is closely intertwined with insulin resistance (IR), overweight, type 2 diabetes mellitus (T2DM), dyslipidemia, hypertension, and hyperglycemia. NAFLD is defined as hepatic fat content >5% of liver weight (steatosis), a potential reversible condition that could evolve into nonalcoholic steatohepatitis (NASH) in 10–25% of subjects, fibrosis, and cirrhosis. It has been reported that NASH patients with advanced fibrosis (F3–F4) were seven times more likely to develop HCC and, in a small proportion, HCC arises in NAFLD individuals without fibrosis [3,4]. Additionally, 30–40% of HCC cases occurred in subjects with cryptogenic cirrhosis, some of which were further affected by dyslipidemia, obesity, T2DM, and possibly by NASH [5]. A study analyzing the surveillance, epidemiology and end results (SEER) registries recorded a 9% annual increase in NAFLD–HCC cases from 2004 to 2009. The survey also examined the prevalence and mortality of 4.929 HCC individuals. Among them, 14.1% of HCC was due to fatty liver, 5% received NAFLD-related liver transplantation (LT), and the presence of NAFLD increased the risk of 1-year mortality, especially in older subjects with previous heart disease [6,7]. Therefore, NAFLD is currently representing not only a clinical and socio-economic burden for health, but it is predicted to overcome HCV, HBV, and alcoholic hepatitis, thus becoming the leading cause of HCC and LT [1,2,3,4,6].

**Figure 1:**
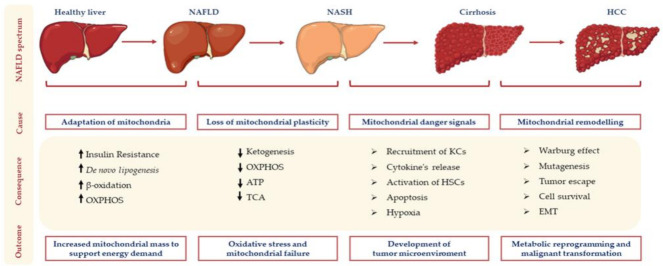
The prominent role of mitochondria in nonalcoholic fatty liver disease (NAFLD)/nonalcoholic steatohepatitis (NASH)-related hepatocellular carcinoma (HCC). Sedentary lifestyle coupled to hypercaloric diet and genetic background lead to the development of insulin resistance (IR), which causes NAFLD onset. In addition, compensatory hyperinsulinemia activates de novo lipogenesis (DNL) and exacerbates hepatocytic fat accumulation. From the early stages of NAFLD, mitochondria adapt in number and function in response to lipid overload, increasing β-oxidation, oxidative phosphorylation (OXPHOS) capacity and biomass (mitochondrial adaptability). Nevertheless, mitochondrial flexibility is compromised during fatty liver progression towards NASH, resulting in blunted ketogenesis, tricarboxylic acid cycle (TCA), OXPHOS and adenosine triphosphate (ATP) production. Consequently, mitochondrial oxidative stress and the release of mitochondrial danger signals worsen inflammation by recruiting and activating Kupffer cells (KCs) alongside fibrogenesis through hepatic stellate cells (HSCs) activation. The inflammatory response together with enhancing apoptosis and hypoxia contribute to generating the surrounding microenvironment that influences malignant transformation and tumor escaping mechanisms. Thus, the loss of mitochondrial dynamics, the accumulation of damaged mitochondria and the remodeling of mitochondrial activities may lead to metabolic reprogramming of hepatocytes, characterized by the switch towards the Warburg effect, mutagenesis, epithelial–mesenchymal transition (EMT) and several strategies of tumor escape from apoptosis in order to promote the compensatory proliferation and HCC onset.

The pathogenesis of NAFLD involves the interplay of environmental, epigenetic, and genetic factors. The outbreak of HCC results from the continuous cycle of parenchymal disruption and tissue regeneration sustained by inflammation, oxidative stress, fibrogenesis and hypoxia. In this scenario, mitochondria, which are extremely adaptable to external cues, play a key role as bioenergetic factories and for the regulation of liver metabolism. Compelling evidence has suggested that mitochondrial dysfunction may precede IR or arise before NASH development, thereby reinforcing the concept that NAFLD may be considered a mitochondrial disorder. Loss of mitochondrial plasticity in terms of functions, morphology and dynamics may support hepatocellular injury and the onset of the Warburg effect, the mechanism by which hepatocytes exploit anaerobic glycolysis even in the presence of oxygen in order to sustain energy demand and cell proliferation [8,9,10,11]. A schematic overview of mitochondrial alterations during NAFLD progression is represented in Figure 1.

Therefore, this review will focus on how environmental factors, genetics/epigenetics and metabolic alterations may impact mitochondrial dysfunction and prime the hepatocytes to epithelial–mesenchymal transition (EMT). The main goal of the present work will be to summarize the main aspects related to NAFLD–HCC pathophysiology driven by mitochondrial failure and the current knowledge about mitochondrial dynamics, which could lay the groundwork for the development of new therapeutic approaches to prevent and/or manage NAFLD progression towards HCC.

## 2. Mitochondria: The Workforce of the Liver

The liver is enriched in mitochondria, highly dynamic organelles endowed with their own mitochondrial DNA (mtDNA) in multiple copies encoding 13 subunits of the electron transport chain (ETC), 22 transfer RNAs, and two ribosomal RNAs [12]. Mitochondria are vital for cell homeostasis and its metabolic activities, as they provide the bulk of energy requirements through oxidative phosphorylation (OXPHOS) and adenosine triphosphate (ATP) synthesis, as well as regulating redox status, β-oxidation, tricarboxylic acid cycle (TCA), ketogenesis and glucidic/lipidic metabolism. Physiologically, mitochondria are renewed from pre-existing ones in a cycle known as mitobiogenesis, encompassing fusion and fission events, since they cannot be generated de novo [12,13]. Recently, our group has proposed an extensive review of mitochondrial dynamics and its involvement in NAFLD pathogenesis [11].

Energy shortage and low ATP availability stimulate hepatic mitobiogenesis by activating peroxisome proliferator-activated receptor (PPAR)-γ coactivators 1 alpha (PGC1α), which are induced by fibroblast growth factor 21 (FGF21) and promote the Krebs cycle, lipid catabolism and gluconeogenesis [14,15]. Notably, Bhalla et al. demonstrated that PGC1α overexpression may promote HCC development by coordinately sustaining mitochondrial biogenesis and β-oxidation [16]. Mitochondrial fusion supports OXPHOS and mitochondrial coupling efficiency during cell proliferation. The elongation of mitochondria is orchestrated by Mitofusin ½ (MFN1, MFN2) and optical atrophy 1 (OPA1), which mediate the merging of mitochondrial outer membranes (MOMs) and inner membranes (IMMs), respectively [17,18,19]. Conversely, during fission, mitochondria are separated into two or more daughter organelles by dynamin-related protein 1 (DRP1), which is recruited around MOMs by specific adaptors such as fission 1 (FIS1), mitochondrial fission factor (Mff) and mitochondrial dynamics (MiD) Proteins 49/50 [20,21,22]. Aberrancies in mitochondrial dynamics are drivers of HCC development and progression. In particular, alterations in MFNs and OPA1 functions may lead to metabolic reprogramming and EMT, while DRP1 de-regulation may prompt cell growth, tumor microenvironment and invasiveness [23].

Mitochondrial dynamics further include a mechanism that allows them to either repair damaged mitochondria through mitochondrial unfolded protein response (UPR^mt^) or definitively disrupt them by autophagy (mitophagy) in order to prevent mitochondrial failure [12]. Mitophagy is fine-tuned and regulated within hepatocytes to the extent that three types of mitophagy participate to preserve cell homeostasis. When mitochondria are preparing to separate, one daughter mitochondrion is transiently hyperpolarized while the other one is hypopolarized. The latter may run into complete depolarization by losing protonmotive force, uncoupling OXPHOS and dissipating mitochondrial membrane potential [20,24]. Suboptimal mitochondria that do not pass the quality check are selected for type 1 mitophagy, which is mediated by phosphatase and tensin homologue (PTEN)-induced kinase 1 (PINK1)/Parkin signaling [20,24,25]. Both fructose and the western diet, exploited as a dietary model of hepatic steatosis, may hasten mitochondrial depolarization and mitophagy, thus giving rise to mitochondrial dysfunction at early stages of NASH, possibly contributing to liver disease progression towards HCC [26]. Type 1 mitophagy is tightly linked to nutrient availability and insulin signaling, while type 2 mitophagy may either occur in parallel with PINK1/Parkin-dependent mitophagy or be induced by photodamage in a phosphoinositide 3-kinase (PI3K)-independent manner [20,24,27]. In both type 1 and 2 mitophagy, E3 ubiquitin ligases are enrolled on MOMs and provide polyubiquitin tails to mitochondrial receptors (i.e., BNIP3, FUNDC1) containing light chain 3 (LC3)-interactive region (LIR) motifs. The ubiquitination of LIR domains is required for binding the autophagic compartments, and deficiency in autophagic processes was associated with mitochondrial dysfunction and genomic instability in murine hepatocytes [27]. Finally, type 3 micromitophagy embedded mitochondrial-derived vesicles (MDVs) containing selected oxidized cargoes and mitochondrial fragments into multivesicular bodies, which then merge into lysosomes for degradation [20,24,28].

## 3. Mitochondrial Alterations at Early Stages of Hepatic Steatosis: Cause or Consequence?

The mechanisms underlying NAFLD pathogenesis are highly complex and multifactorial, and parallel factors participate in the disease onset and progression. A sedentary lifestyle and hypercaloric diet are the major risk factors that influence visceral adiposity and the development of peripheral IR. It has been reported that defects in mitochondrial biogenesis may rapidly accelerate beige-to-white adipocytes transition, thus contributing to adipose tissue expansion [29]. Benador et al. revealed that the mitochondria surrounding lipid droplets (LDs) in the adipose tissue, termed peri-droplet mitochondria (PDM), showed a different bioenergetic metabolism compared to those non-adjacent to the LDs surface, thus contributing to their enlargement and adiposity [28]. Furthermore, signs of hepatic mitochondrial dysfunction have been observed in insulin-resistant rats without overt NAFLD, thereby suggesting that mitochondrial failure may appear as an early event before steatosis onset [30].

Fatty liver results from the unbalance between lipid synthesis and catabolism within the hepatocytes. IR promotes adipose tissue lipolysis, which consequently causes an efflux of free fatty acids (FFAs) to the liver, where they are stored as triglycerides (TGs) to counteract the harmful effect of FFA surplus. In addition, the compensatory hyperinsulinemia activates the hepatic de novo lipogenesis (DNL) through sterol regulatory element-binding proteins (SREBP1, SREBP2), ATP-citrate lyase (ACLY), acetyl-CoA carboxylase (ACC) and fatty acid synthase (FASN), which provide precursors for TG synthesis, thus exacerbating LDs accumulation [31]. In response to high caloric intake, IR and obesity, hepatic mitochondria adapt in number, biomass and activity through a mechanism known as “mitochondrial flexibility”. Firstly, the liver increases FFA transport into mitochondria through carnitine palmitoyltransferase-1/2 (CPT1/2) and enhances both β-oxidation and OXPHOS. Indeed, megamitochondria were observed in liver biopsies of both NAFLD and NASH patients, suggestive of an enhanced mitochondrial density [32]. Shami and colleagues have recently proposed a detailed description of the three-dimensional ultrastructure of giant mitochondria in NAFLD subjects. The authors provided a classification of these organelles, distinguishing elongated, irregular, and spheroidal shapes based on the characterization of their internal ultrastructure [33], although further investigations are required to elucidate whether the rise in mitochondrial content observed in NAFLD/NASH hepatic tissues reflects high levels of mitobiogenesis or reduced mitophagy [34]. Then, FFA overload overwhelms both TCA and FA catabolism, from which NADH/FADH_2_ are generated. Both NADH and FADH_2_ transfer electrons to the respiratory chain coupled to the synthesis of ATP, and a small fraction of protons leak from ETC, react with oxygen (O_2_), and generate reactive oxygen species (ROS) [35]. Elevated concentrations of lipid species, especially those incorporating saturated/monounsaturated FA chains, critically promote both ROS-induced lipotoxicity and hepatocellular damage, thus favoring liver disease progression and loss of mitochondrial dynamics [35,36]. Therefore, this section will discuss how lipid metabolism, in terms of composition, anabolism and catabolism, may impact mitochondrial dysfunction and favor per se a pro-tumorigenic microenvironment from the early stages of NAFLD.

### 3.1. Fatty Acids Metabolism, De Novo Lipogenesis and β-Oxidation: From Steatosis to HCC

NAFLD development is directly involved in hepatocarcinogenesis and tumor adaptation to local micro-environment independently of NASH. The most studied mechanisms linked to carcinogenesis affect mitochondria and include the Warburg effect, from which lactate and pyruvate are produced to support energy demand [8,9,11], and glutaminolysis, which exploits glutamine to sustain the Krebs cycle by generating a high citrate concentration [37]. These facts notwithstanding, the aberrant activation of DNL is one of the major metabolic events occurring in NAFLD–HCC onset. In this field, the role of lipid species is attracting increasing attention, as they can modify mitochondrial functionality and contribute to metabolic switching in hepatocytes, to the extent that alterations in lipid metabolism are currently recognized as a hallmark of hepatic cancer.

Many malignant tumors showed LDs accumulation and the activation of lipogenic pathways, which were correlated with the pro-survival phenotype in human HCC cell lines and poor prognosis in HCC patients [38]. Among lipid classes, TGs, diglycerides and ceramides are enriched in steatotic livers and contribute to the onset of endoplasmic reticulum (ER) stress, whose alterations in terms of functions and architecture participate in both DNL and IR [39]. It has been demonstrated that lipid overload may disrupt ER–mitochondrial communications in steatotic hepatocytes and *ob*/*ob* mice fed a high-fat diet (HFD). Lipids altered the abundance of calcium (Ca^2+^) transporters and channels leading to Ca^2+^ efflux into the cytoplasm. The increased intracellular Ca^2+^ concentration enhanced ROS content and the mutagenesis of both nuclear DNA and mtDNA, thereby inducing the activation of oncogenes or the inhibition of onco-suppressors, and affecting mtDNA replication [40,41].

Several studies revealed that aberrantly activated DNL is critical for HCC development and progression. The inhibition of stearoyl-CoA desaturase (SCD), FASN and ACC, which provide FFAs within hepatocytes, abrogated Akt-driven HCC and reduced the hepatic cancer stem cells (CSC) pool [42,43]. Consistently, FFAs per se derived from adipose tissue lipolysis, combined with those newly synthetized from DNL, may hasten hepatocytes degeneration and foster mechanisms of tumor escape by activating anti-apoptotic programs. FFAs may be converted into mono-unsatured fatty acids (MUFAs) by desaturase enzymes as fatty acid desaturase (FADS) and SCD. Increased MUFAs, Fads1/2 and Scd2 levels have been observed in mice affected by NAFLD–HCC and human HCC specimens [40,44,45]. Moreover, rat hepatocytes treated with palmitic acid (PA) affected insulin signaling, enhanced β-oxidation rate and exacerbated ROS content. PA also activated the c-Jun NH2-terminal kinase (JNK), which is the most constitutively activated factor in HCC involved in mitochondrial cytochrome c release, cell death, and compensatory proliferation [46,47]. Kudo et al. found that hepatocyte-specific *Pik3ca* transgenic mice, a genetic model of hepatosteatosis, developed hepatocellular adenomas with abundant LDs and HCC, but both without inflammation and fibrosis, supporting a direct role of lipids as pro-tumorigenic factors. Notably, they demonstrated that the accumulation of oleic acid (OA) and PA promoted liver cancer development by suppressing Pten, an inhibitor of Pi3k/Akt/mammalian target of rapamycin (mTOR) signaling [44].

In response to an excessive FFA concentration, mitochondria enhance CPT1/2-mediated mitochondrial lipid flux and β-oxidation to protect against lipotoxicity, a mechanism that is lost during the NAFLD course. Moreover, uncoupling protein 2 (UCP2) activity is upregulated due to FA surplus, thus boosting mitochondrial proton leak and increasing NASH susceptibility [35]. Defects in the mitochondrial trifunctional protein (MTP), which catalyzes long-chain FA β-oxidation, induced hepatic IR and steatosis development, alongside an increase in antioxidant defenses and cytochrome P-450 to counteract ROS production [45]. Reduced FA catabolism, ATP and mitochondrial membrane potential have been reported in progressive NAFLD and in many NAFLD–HCC cases. Diethylnitrosamine (DEN)-injected mice fed with HFD downregulated both CPT2 and β-oxidation during HCC onset. The suppression of FA oxidation enables HCC cells to adapt to a lipid-rich microenvironment and to escape lipotoxicity by blocking JNK [48]. CPT2 reduction results in the accumulation of acylcarnitine species, which further hamper β-oxidation and facilitate the acquisition of stem cell properties through the signal transducer and activator of transcription 3 (STAT3) [48,49].

However, T2DM, obesity or NAFLD etiologies may contribute to the development of “oxidative” HCCs, a metabolic HCC variant observed in a subset of patients, as these conditions provide FFAs in bulk that, in turn, may be re-routed towards β-oxidation rather than TG re-esterification [11,49,50,51,52]. In this context, PPARα, the master regulator of FFA catabolism, activates and stimulates the WNT/β-catenin oncogenic cascade which, in turn, sustains FFA dismissal. Additionally, β-oxidation is further supported by either the hydrolysis of intrahepatic TGs, which provide FFA substrates, or acetyl-CoA, which derives from β-oxidation and promotes ketogenesis. In “oxidative” HCCs, FFA degradation efficiently feeds the ETC and supplies ATP, thus rendering its synthesis dependent on FFAs oxidation at the expense of ATP produced by the Warburg effect [52].

### 3.2. The Role of LDs and Lipophagy in NAFLD-Related HCC

LDs, whose biogenesis starts from ER-Golgi compartments, are highly dynamic organelles stocking energy sources and working as a buffering system incorporating lipotoxic species. LDs may either re-arrange in size according to nutritional status, or support cell survival by providing FFAs via autophagic processes during stressful conditions. A computational analysis performed in 11 human HCC tissues highlighted that the majority of HCCs reduced FFA uptake and β-oxidation, despite the fact that TG content was enormously raised compared to its non-tumoral counterpart. In in vitro models of hepatic steatosis, our group demonstrated that the presence of a saturated lipid profile, including TG species, was associated with an aggressive hepatocellular phenotype mimicking HCC [10]. Consistently, a lipidomic analysis evaluating 20 HCC tissues revealed that levels of TGs with multiple double bonds were downregulated, while saturated TG species were greatly overexpressed [53]. Berndt et al. suggested that TG content in HCCs does not reflect the rate of FFA uptake, but it was determined by the combination of FFA esterification and the degradation of lipases hydrolyzing LDs [51]. Tumor cells largely exploit LDs breakdown in the absence of energy availability and hypoxia to support cell growth and tumor expansion [54,55,56,57]. It has been shown by Tian et al. that unbalanced lipophagy is unexpectedly involved in carcinogenesis in hepatoma cell lines, NAFLD murine models, and NAFLD–HCC human samples [58]. Specifically, Nogo-B oncogene, which localizes at the ER–LDs interface, interacts with autophagy related 5 (ATG5) to promote LDs self-catabolism. LDs’ breakdown releases lysophosphatidic acid (LPA) mediator, which enhances the pro-proliferative Hippo/Yes-associated protein (YAP) cascade [58]. Moreover, oxidized low-density lipoprotein (oxLDL) and free cholesterol accumulate in the hepatic microenvironment of NASH mice [58] and NAFLD subjects [59]. The increased uptake of oxLDL in the liver via CD36 receptor may represent a triggering stimulus for the metabolic rewiring of the hepatocytes, as it can promote Nogo-B transcriptional activation [58].

Furthermore, in both human hepatocytes laden with large LDs and cirrhotic scars flanked by macrovescicular steatosis, the nuclear localization of the YAP protein was increased compared to hepatocytes accumulating small LDs and cirrhotic sections with micro-steatosis [60], suggesting a link between macro-steatosis and cancer. Conversely, the characterization of the LDs proteome highlighted that perilipin (PLIN1), ADRP (PLIN2) and TIP47 (PLIN3) proteins mediate LDs–mitochondria crosstalk, thus regulating LDs’ expansion and disposal. Notably, PLIN1, PLIN2 and PLIN3 are differentially expressed during tumorigenesis and usually dwell on the LDs’ surface according to the LDs’ dimension. PLIN2 and PLIN3 mainly coat small LDs and are commonly overexpressed at early stages of HCC, as the LD dimension allows rapid dynamics between synthesis and consumption to sustain phases of cell proliferation or metabolism. PLIN1 expression is lost during hepatocarcinogenesis and may reflect the differentiation grade of hepatocytes [61]. Th eactivation of SREBP1 via PI3K/Akt/mTOR further contributes to neoplastic steatogenesis and PLINs expression [38]. These studies pointed out a differential role of micro/macro-LDs in HCC onset and progression, although details on the mechanisms and respective roles need to be addressed in the future.

## 4. Mitochondria Play a Crucial Role in the Switch from NASH towards HCC

A continuous flux of FFAs into mitochondria results in increased oxidative damage associated with mitochondrial dysfunction, ER stress and tissue inflammation, which may contribute to the progression from NAFLD to NASH, and up to HCC [60]. Over the course of NAFLD, mitochondrial failure in terms of functions, morphology and dynamics occurs in attempting to deal with energy surplus and to protect against FA-induced lipotoxicity, with the consequent loss of mitochondrial plasticity. In both mice and humans affected by NASH, blunted ketogenesis and mitochondrial respiration were observed, whereas the citric acid cycle was increased in the attempt to discard lipid overload [61].

Compromised OXPHOS, ketogenesis, low ATP synthesis and incomplete β-oxidation coupled to an overreactive Krebs cycle incremented ROS production, causing mitochondrial abnormalities, lipid peroxidation and mtDNA damage. Increased serum levels of malondialdehyde (MDA), a byproduct derived from the oxidative degradation of lipids, were detected in NAFLD patients, while antioxidants coenzyme Q10 and CuZn-superoxide dismutase (SOD) were reduced [62]. The mitochondrial ROS production triggers mitogen-activated protein kinases (MAPKs) and induces JNK phosphorylation (p-JNK), affecting the mitochondrial ETC and damaging ROS production [63]. Interestingly, if on one hand p-JNK may antagonize oxidative damage by enhancing apoptosis, on the other hand, the signals arising from necrotic hepatocytes may stimulate the JNK cascade in Kupffer cells (KCs) in order to express the pro-tumorigenic cytokines interleukin 6 (IL-6) and tumor necrosis factor-alpha (TNF-α), which promote EMT, migration and invasiveness [62].

ROS induce the activation of 5′ AMP-activated protein kinase (AMPK), which prompts PGC1α. The latter is a powerful sensor of nutritional status that is activated in response to fasting, glucocorticoids and dietary FFAs, and it plays a crucial in role in the regulation of OXPHOS, mitobiogenesis and glucidic/lipidic metabolism [14]. The PI3K/Akt/mTOR cascade mediates PGC1α activation, and in turn induces transcriptional factors (i.e., PPARs, nuclear respiratory factors (NRF1/2,), estrogen-related receptors) that increase the levels of ROS scavengers, including SOD2 and glutathione peroxidase 1 (GPX1). However, antioxidant defenses are not able to manage the long-term ROS exposure during NASH. Indeed, lower levels of SOD2 were observed in both HFD-fed rodents and humans [63], while higher circulating levels of the glutathione disulfide/glutathione (GSSG/GSH) ratio and glutathione transferase (GST) were found in a small cohort of 21 pediatric NASH patients, as a possible compensatory mechanism against oxidative stress [64].

In response to mtDNA, proteins and lipids damage induced by oxygen radicals, the nicotinamide adenine dinucleotide (NAD^+^)-dependent histone deacetylase sirtuins (SIRTs), localizing on mitochondria, promote SOD2 activation through its deacetylation. In NAFLD subjects, SIRT1, SIRT3, SIRT5 and SIRT6 are downregulated, while SIRT4 was overexpressed in response to the exacerbated DNL [65]. Moreover, several members of the SIRT family may alleviate ROS-induced hepatocellular injury and promote apoptosis, thus preventing compensatory proliferation and tumorigenesis [66,67].

NASH is characterized by the loss of mitochondrial flexibility, the activation of Kupffer cells (KCs), hepatic macrophages, and even by the presence of fibrosis. Cytokines released by both hepatocytes and KCs may activate hepatic stellate cells (HSCs), which produce collagen and fibrotic scars, and further stimulate apoptotic receptors, such as Fas, TNF receptor 1 (TNFR1) and TNF-related apoptosis-inducing ligand (TRAIL) on hepatocytes surface [67,68,69]. Consequently, the pro-apoptotic B-cell lymphoma 2 (Bcl-2) and JNK pathways stimulate the mitochondrial membrane’s permeabilization through their translocation on MOMs, where they can create tunnels, from which apoptosis-inducing factor (AIF) and cytochrome c are released, promoting mitochondrial derangement and apoptosis [69]

Furthermore, the low rate of mitophagy in NAFLD hampers the disruption of degenerative mitochondria, causing the release of mitochondrial damage-associated molecular patterns (mito-DAMPs) that even trigger inflammation. Mito-DAMPs may prompt pattern-recognition receptors (PRRs) by binding and activating the Toll-like receptor 4/9 (TLR4/9) plus the downstream nuclear factor kappa-light-chain-enhancer of activated B cells (Nf-κB) signaling, the nucleotide-binding oligomerization domain (NOD)-like receptors (NLRs), the pyrin domain containing 3 (NLRP3) inflammasome and interferon regulatory factor-dependent type 1 (IRF1) [68]. Ping et al. demonstrated that the presence of hepatocytes-derived mtDNA, secreted from damaged liver tissues, in the circulation triggers the fibrotic response, by interacting with TLR9 on KCs and inducing collagen deposition by activated HSCs [69].

**Figure 2:**
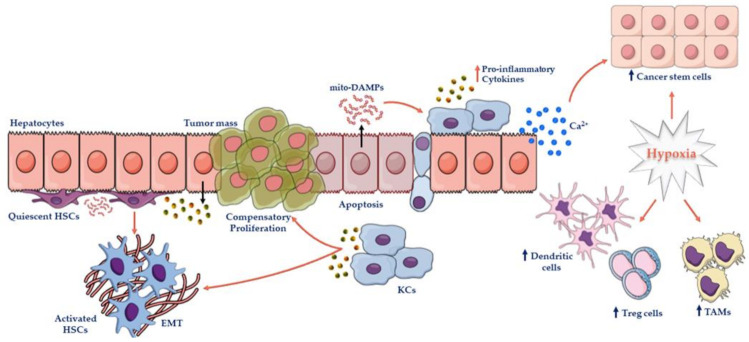
The landscape of HCC microenvironment in NAFLD/NASH. Over NAFLD progression, ER/oxidative stress, loss of mitochondrial adaptability and mitochondrial failure activate hepatocellular apoptotic and inflammatory pathways. Hepatocytes release mitochondrial damage-associated molecular patterns (mito-DAMPs) derived from damaged mitochondria. Mito-DAMPs bind pattern-recognition receptors (PRRs) on the Kupffer cells’ (KCs) and HSCs’ surface, exacerbating tissue inflammation and fibrosis. In addition, HSCs’ activation may also be stimulated by pro-inflammatory cytokines released from both hepatocytes and KCs. In turn, KCs enhance pro-proliferative signals (i.e., IL-6), thus triggering the compensatory proliferation of hepatocytes, a possible mechanism to avoid apoptosis. The continuous exposure to inflammation, fibrosis, and apoptotic signals worsens hepatic oxygen distribution (hypoxia). Hypoxia together with increased Ca^2+^ efflux, derived from the disruption of ER–mitochondrial communications, promote the growth of CSCs and the recruitment of tumor-associated macrophages (TAMs), T-regulatory cells (Treg) and dendritic cells, which suppress cytotoxic immune response and co-adjuvate hepatocarcinogenesis.

Although mitochondrial dysfunction plays a prominent role in NAFLD progression, ER stress also contributes to the severity of liver damage. Indeed, these two organelles physically communicate through mitochondrial-associated membranes (MAMs) in response to stress conditions [70]. MAMs are required for the transport of lipids, Ca^2+^, insulin signaling and glucose homeostasis, and even for the interplay with the UPR, which is considered a sensor of ER stress [71]. As previously mentioned, fat accumulation may disturb the ER–mitochondria network, causing either Ca^2+^-induced apoptosis or DNA injury. Additionally, cytosolic Ca^2+^ may contribute to hepatocarcinogenesis by activating the compensatory proliferation of hepatic CSCs (Figure 2) [70,71]. Therefore, this section aims to delineate the contribution of mitochondrial dysfunction and the loss of mitochondrial flexibility to the progression from simple steatosis to NASH, and how these abnormalities may generate an advantageous microenvironment for tumorigenesis.

### 4.1. The Loss of “Mitochondrial Flexibility” during NASH May Play a Role in HCC Development

As previously underlined, the derangement of mitochondrial adaptability, through which mitochondria modify their function and number in response to nutrients availability, may drive and/or accelerate NASH development and its progressive forms, despite further studies being required to elucidate which mechanisms among fusion, fission or mitophagy most influence the natural history of NAFLD [32,72].

The disequilibrium of mitobiogenesis and the accumulation of damaged mitochondria, mainly due to the failure of mitophagy, have been observed in the liver tissues of several NASH subjects, and NASH has even been correlated with alterations in mitochondrial architecture [73]. In murine models, the genetic deletion of *Mfn2*, which is involved in mitochondrial elongation during fusion, led to mitochondrial failure, ER stress and higher levels of H_2_O_2_ [74]. Contrariwise, the administration of omega-3 polyunsaturated FAs (PUFAs) in HepG2 cells, previously incubated with PA and OA to mimic steatosis, increased the expression of *Mfn2* with the consequent elongation of mitochondrial tubules [75]. It has been observed that *Mfn1*-deficent mice displayed an increase in *Mfn2* and *Opa1*, as a possible compensatory mechanism. Higher levels of *Opa1* promote the remodeling of mitochondrial cristae and avoid apoptosis by inhibiting cytochrome c release [72]. Fibrotic mice fed with a high-trans-fat, high-fructose and high-cholesterol (AMLN) diet showed an increased number of disrupted mitochondria, accompanied by reduced OXPHOS capacity, and the loss of both mitochondrial integrity and cristae structure. In these rodents, the expression of *Mnf1* and *Opa1* was significantly reduced, and they reflected a higher number of separated mitochondria [76]. Recently, Zahng et al. demonstrated that *Mnf1* expression was reduced in 34 HCC patients. To explore the role of mitochondrial fusion, they exploited an in vitro model using an MHCC97-H cell line and observed that *Mnf1* inhibits cell proliferation, migration and invasion. These results suggest that the loss of mitochondrial dynamics, mainly due to the deletion of *Mnf1*, plays a crucial role in impeding HCC development [77].

Regarding alterations in mitochondrial fission, it has been remarked that the deletion of *Drp1* in mice inhibited Bcl-2 and the release of cytochrome c [20]. In mice challenged with HFD, lacking the *Drp1* gene ameliorated hepatic fat content and ER stress through the expression of Fgf21, which plays a beneficial role in mitochondrial dynamics and prevents the release of pro-fibrotic mediators, suggesting that the inhibition of mitochondrial separation may improve NAFLD severity [78]. Nevertheless, Pollard et al. generated a liver-specific murine model in which AMPK and fission were constitutively activated (iAMPK^CA^). Chronic mitochondrial scission protected against obesity, steatosis, necroinflammation and fibrosis, as it swiftly drove damaged mitochondria into the autophagic compartments [79].

### 4.2. Megamitochondria and Mitophagy: The Impact of Morphological Alterations in NASH

Fusion–fission unbalancing affects mitochondrial architecture and promotes the formation of megamitochondria, which were detected in both adult and pediatric NAFLD/NASH patients [32,33]. In a cohort of 31 biopsied NASH patients, megamitochondria localized in the central lobule adjacent to the central vein and the portal triads have been, and these featured crystalline insertions [80]. In another study, liver biopsies of NASH patients showed an alteration of mitochondrial morphology characterized by megamitochondria, cristae paucity and opacity of granules [81]. Ahishali and colleagues performed quantitative and semi-quantitative ultrastructural evaluations of liver biopsies from 23 patients, 10 with NAFLD and 13 with NASH [82]. Both NAFLD and NASH hepatic tissues were characterized by the presence of megamitochondria with no significant differences among the two groups. However, NASH patients, much more so than NAFLD ones, showed higher mitochondrial diameters, intra-mitochondria crystalline inclusions and granules in the matrix, which correlated with both mitochondrial swelling and OXPHOS failure [80]. Recently, Verhaegh et al. observed ultrastructural changes by using transmission electron microscopy in 37 NAFLD patients, of whom 12 had NASH [83]. In this study, the presence of giant mitochondria showed no differences between patients with or without NASH, according to the results of Ahishali et al. [82], suggesting that the appearance of megamitochondria could represent a transitory phase between simple steatosis and NASH. However, their exact role in NAFLD pathophysiology needs to be clarified.

The presence of giant and/or globular mitochondria in fatty liver has been associated with the suppression of mitophagy to the extent that restoring mitophagy may enable the rescuing of mitochondrial dysfunction in NAFLD [84]. Mice carrying a *Parkin* deficiency, which is involved in type 1 mitophagy, displayed swollen mitochondria with loss of cristae [85]. Moreover, the high caloric intake in these mice reduced the expression of LC3 receptors, with the consequent activation of inflammatory response, which leads to the inhibition of mitophagy and NASH progression [86]. Glick and colleagues exploited two murine models deleted for *Parkin* or *BCL2/adenovirus E1B 19 kDa–protein-interacting protein 3* (*Bnip3*) genes, in which lipid synthesis was exacerbated, possibly developing a NASH phenotype. In these rodents, the authors observed the presence of globular mitochondria and unidentifiable cristae [87]. Moreover, changes in mitochondrial ultrastructure were evaluated in a mouse model of NASH, in which the CXCR3 factor was strongly upregulated. The presence of CXCR3 promotes inflammation in chronic liver disease and may impact mitochondrial morphology, which appear round shaped with disrupted cristae. On the contrary, the deletion of the *CXCR3* gene ameliorated mitochondrial architecture in terms of less swollen mitochondria and more organized cristae, suggesting that its ablation could prevent morphological alterations [88].

Finally, the lipidomic analysis of NASH patients revealed higher hepatic levels of dihydroceramide and dihexosylceramide species that were correlated to defects of mitophagy, oxidative stress and inflammation [89]. The accumulation of dihexosylceramide has even been found in human HCC tissue, suggesting that reduced mitophagy may be involved in the progression from NASH to HCC [90].

### 4.3. The Contribution of Hepatocellular Mitochondrial Dysfunction and Inflammatory Response to NASH

In the large spectrum of NAFLD pathogenesis, inflammation and apoptosis are benchmarks of NASH progression. De-regulated mitochondrial activity within hepatic cells and hepatocytes-derived danger signals may directly or indirectly induce inflammation and apoptosis, precipitating fibrosis, cirrhosis and, eventually, HCC development. Clinical and experimental evidence has showed that ROS-induced apoptosis triggers TRAIL receptors that, subsequently, promote the release of cytokines and chemokines [68]. Nevertheless, the role of TRAIL is controversial. TRAIL^−/−^ mice showed worsened inflammation and fibrosis but improved adipose tissue injury, suggesting that TRAIL is indispensable for adipose tissue homeostasis but promotes the hepatic inflammatory and fibrotic response [69]. A common defect observed in obesity and NAFLD/NASH is cardiolipin peroxidation, a mitochondrial phospholipid that regulates mitochondrial dynamics and morphology. Pathological cardiolipin species may be generated by either acyl-coA lysocardiolipin acyltransferase 1 (ALCAT1) or oxidative stress, causing cardiolipin exposure to MOMS of damaged mitochondria [91]. The externalization of oxidated cardiolipin activates apoptotic processes by inducing cytochrome c release [92], and represents a mitophagic signal to induce mitochondrial dismissal [11]. Cardiolipin inhibitors could ameliorate NAFLD pathogenic manifestations by modulating NLRP3 inflammasome and KCs recruitment [93]. It is noteworthy that cardiolipin is frequently downregulated during HCC progression as a possible strategy to avoid apoptosis [92].

Apoptotic signals impaired mitochondrial biogenesis as they dampened mitophagy and induced the release of mito-DAMPs. Many components of mitochondria, such as formyl peptides, share structural similarities with bacteria and could allow cell-to-cell communications by binding the PRRs expressed on the surface of KCs and HSCs, thus exacerbating the systemic secretion of pro-inflammatory TNF-α, IL-6, IL-1β and IL-8 (Figure 2) [94,95,96]. Among mito-DAMPS, mtDNA represents the major active components released by damaged hepatocytes, and may interact with TLR9, thereby activating inflammation and fibrosis [41]. Both mtDNA and formyl peptides could recruit and induce the migration of polymorphonuclear neutrophils through MAPKs, thus eliciting neutrophil-mediated hepatic injury [97]. Consistently, elevated concentrations of mito-DAMPs were detected in HFD-fed mice and serum samples of NAFLD/NASH patients, especially in those with advanced fibrosis. Moreover, circulating levels of mtDNA reflected hepatocellular injury, and were associated with histological parameters of disease progression in both rodents and humans [41,98].

Inflammasome activation is closely linked to progressive NAFLD, and its expression was higher in both NASH mice and patients [70]. TLRs, TNFR1 and harmful stimuli, including mito-DAMPs, increased intracellular levels of ATP and Ca^2+^ ions as well mitochondrial-derived ROS, and activate NLRP3 inflammasome and co-adjuvate its assembly. Interestingly, excessive mitochondrial fission at the expense of fusion has been associated with NLRP3 activity under hyperglycemic and diabetic conditions [99]. Wei and collaborators first demonstrated that the expression of NLP3 inflammasome was completely lost or significantly downregulated in 128 HCC patients. NLRP3 promoted not only the activation of inflammatory pathways, but also the reestablishment of hepatic homeostasis, by activating apoptosis. Therefore, a complete deficiency of NLRP3 favored the compensatory proliferation of hepatocytes and HCC onset [100]. Conversely, NLRP2, belonging to the inflammasome cascade as an inhibitor of NF-kB signaling, may participate in NASH improvement. The suppression of the *Nlrp2* gene accelerated steatosis development in mice. The hepatic expression of NLRP2 was also found to be lower in NASH subjects, with a consequent increase in IL-1β and IL-18 caused by KCs [71].

The mito-DAMPs-induced inflammation, combined with mitochondrial oxidative damage within hepatocytes, further modulates the efficacy of nuclear receptors, such as liver X receptors (LXRα, LXRβ) and members of the PPAR family (PPARα, PPARβ, PPARγ), which are key regulators of mitochondrial activity (i.e., β-oxidation) and may be crucial in NASH progression. In particular, LXRs’ downregulation may sustain fat accumulation by increasing SREBP1-c and reducing the very low-density lipoprotein (VLDL) catabolism [101]. The administration of SR9238, which is an agonist of LXR, is able to significantly reduce hepatic inflammation in NASH mice [102,103]. Moreover, Pparγ-deficient mice displayed higher levels of TNF-α, suggesting that PPARγ may regulate the KCs-mediated inflammatory response [104]. Likewise, PPARα blocks NF-κB and oxidative burst, and it prevents the progression towards fibrosis [105,106]. Mice fed a choline-deficient, ethionine-supplemented (CDE) diet showed increased hepatic TG content and developed NASH alongside reduced PPARs and PGC1α, showing lower levels of mitochondrial mitobiogenesis [107]. Additionally, in PGC1α^−/−^ mice exposed to an obesogenic diet, Besse-Patin et al. correlated the inefficient β-oxidation and ROS production with a fibrotic response, suggesting that mitochondrial damage in hepatocytes may directly aggravate the NASH condition [108]. In a cohort of 85 biopsied patients, it has been found that a lower expression of PPARα correlated with the severity of liver disease, suggesting that it may represent a possible pharmacological target in NASH treatment [109].

Finally, some have postulated an interplay between PPARα and hepatocytes proliferation. The oncogene Cyclin D1 inhibits its expression, and consequently reduces the levels of β-oxidation in HCC cell lines, confirming that PPARα is even involved in carcinogenesis [110]. Ergo, such findings provide an overview of the cross-talk among mitochondrial dysfunction occurring in hepatocytes and activated inflammatory response, highlighting the relevance of mitochondria dysregulation in active NASH.

### 4.4. The Crosstalk among Parenchymal Mito-DAMPs, HSCs and Inflammation in NASH-Driven HCC

As discussed above, mitochondrial dysfunctions activate both KCs and HSCs, which, in turn, exacerbate inflammation and promote fibrotic response, respectively. The ROS production, lipid peroxidation, mito-DAMPs and cell death signals activating caspases augment the hepatic infiltration of pro-inflammatory cells, and together trigger fibrogenesis (Figure 2) [111,112]. In particular, 4-hydroxynonenal (4-HNE), an end-product of lipid peroxidation, acts as a potent pro-fibrogenic stimulus for the expression of genes involved in extracellular matrix deposition, the primary mechanism of fibrosis and cirrhosis [113,114]. HSCs can be activated by the cytokines and chemokines, such as TNFα, IL-1β, IL-6, TGF-β, and C-C Motif Chemokine Ligand (CCL) 2 and 5, released from hepatocytes and KCs. In turn, KCs may enhance the production of chemoattractant factors and ROS by NADPH oxidase, and further worsen the inflammatory response and HSCs-activated phenotypes. Recently, Ping et al. demonstrated that the chronic administration of hepatotoxic thioacetamide (TAA) in mice for 6 weeks can cause hepatocytic cell death along with hepatic collagen accumulation. According to these findings, the upregulation of pro-fibrotic genes, such as TGFβ1, pro-collagen α1 and Tissue inhibitor of metalloproteinases-1 (TIMP-1), were detected. TAA-induced liver fibrosis was further correlated with the high levels of circulating mtDNA arising from death hepatocytes. Notably, freshly isolated HSCs exposed to mito-DAMPs assumed morphological features of their activation, with the loss of LDs, a myofibroblast-like phenotype and a robust positivity to alpha-smooth muscle actin (α-SMA), a marker of HSCs activation [98]. In two independent cohorts of NAFLD subjects, it has been observed that mtDNA levels were higher in NASH patients, and much higher in those with fibrosis, compared to NAFLD subjects and matched controls, according to the results obtained in mice [98].

Pro-inflammatory pathways activated by mitochondrial dysfunction and oxidized mtDNA contribute not only to fibrosis, but also to HCC onset. For instance, it has been demonstrated that the NF-κB cascade is involved in cell survival [115]. Park et al. demonstrated that the TNF-α and IL-6 produced by KCs may activate pro-oncogenic pathways via JNK signal transducer and activator of STAT3, Janus kinase 2 (JAK2), MAPK, and PI3K. In dietary and genetic models of hepatosteatosis, it has been shown that IL-6 induced STAT3 activation and triggered hepatocyte proliferation, leading to HCC [116]. Uysal and coworkers proposed a panel of circulating markers of liver damage, including proinflammatory cytokines (TNF-α, IL-6, IL-8), ferritin, nitric oxide and mediators of mitochondrial damage (i.e., MDA), which when used in clinics may predict NASH progression to HCC [117]. Finally, the upregulation of hypoxia inducible factor 1-alpha (HIF-1α) occurs in response to intrahepatic ROS production. HIF-1α is stable under hypoxia, while it is degraded under normal oxygen conditions (normoxia). In NASH, oxygen is unevenly distributed in the hepatic lobules due to both inflammation and fibrotic scars, thus promoting hypoxia. Interestingly, non-tumoral cells such as mesenchymal stem/stromal cells (MSCs) are typically recruited to the injured or hypoxic area. In mice treated with DEN and exposed to a high-fat, high-cholesterol and high-sugar diet (HF-HC-HSD), which triggers liver fibrosis, it has been observed that the increase in HIF-1α coupled with the release in inflammatory cytokines mediated metabolic reprogramming, angiogenesis and proliferation, thereby prompting switching from NASH to HCC [118,119].

### 4.5. “Evasion” from Mitochondrial-Induced Apoptosis Drives HCC

The development of HCC is almost never a sporadic event, but rather a Darwinian selection of those clonal cells able to trigger mechanisms of escape from intrinsic cues, and to adapt to exogenous restraints imposed by the environment. During NASH, lipid peroxidation, mito-DAMPs, oxidative/ER stress and inflammation activate apoptotic receptors, such as FAS, TNFR1 and TRAIL, resulting in parenchymal disruption and regeneration through fibrotic scars (Figure 2) [120]. The infiltration of pro-inflammatory cells and the presence of apoptotic bodies have been observed in the liver of several NASH patients [121]. Although NASH progression is characterized by an extensive apoptosis, non-hepatocytes and non-cellular components (such as the extracellular matrix) allow the generation of the tumor microenvironment, in which hepatic stem cell progenitors may carry out strategies to elude cell death and induce compensatory proliferation [122]. Recently, Anstee and colleagues proposed a review to provide a detailed description of the key NAFLD/NASH mechanisms that promote HCC-evading programs [120].

Inami and collaborators revealed that the liver-specific loss of autophagy is crucial for hepatocellular survival. From the early stages of NAFLD, FFAs and TG overload inhibits autophagy through the activation of mTOR. The abundance of lipid species induces oxidative stress that may also promote the activation of p62, involved in the p62–KEAP1–NRF2 pathway. Phosphorylated p62 triggers NFR2 expression which, in turn, stimulates antioxidant defences [123,124,125]. The improvement of mitochondrial ROS production through p62–KEAP1–NRF2 signaling may represent one of the pro-survival events allowing tumor initiation. The activation of the KEAP1–NRF2 pathway has been reported in more 25% of HCC subjects, and the inhibition of autophagic proteins such as ATG7 and beclin1 may provide an escaping strategy from ROS-induced apoptosis [126,127,128].

Moreover, mitochondrial oxidative stress altered MAMs and incremented the ER-induced efflux of Ca^2+^, thus contributing to hepatocytic cell death. HFD-fed mice treated with cyclosporin A, which inhibited mitochondrial permeabilization by regulating Ca^2+^ turnover, displayed lower levels of apoptosis [129]. Guerra et al. examined the contribution of Ca^2+^ signaling in HCC pathogenesis. The authors exploited HCC cell lines, murine models and human liver samples to investigate the role of the inositol 1,4,5-triphosphate (InsP3) receptor that acts as Ca^2+^ channel, and observed that its release was linked to the proliferation of both hepatocytes and tumor cells. Such findings may suggest that the inhibition of Ca^2+^ entry into mitochondria may sustain tumorigenesis as an escaping mechanism of apoptosis [130].

Interestingly, Huang et al. demonstrated that even the defects in mitochondrial biogenesis during NASH may enhance anti-apoptotic signals and favor cell growth [131]. In human HCC cell lines, such as Bel7402 and SMMC7721, *MFN1* genetic ablation increased mitochondrial fission, leading to ROS generation and ROS-induced mutagenesis, such as the constitutive activation of the PI3K/Akt/mTOR network. Akt drives the downstream degradation of the *TP53* onco-suppressor gene via the E3 ubiquitin–protein ligase MDM2 and, on the other hand, promotes the transcriptional activity of Nf-κB and inhibits autophagy. Indeed, increased levels of the PI3K/Akt/mTOR axis have been observed in about 40–50% of HCC patients [132,133].

Long-lasting exposure to inflammatory response and oxidative burst represents the major force that encourages genomic instability, sensitizing hepatocytes to JAK/STAT, ERK and MAPK growing signals. Cytokines, such as TNF-α and IL-6, were correlated with carcinogenesis and cell division. In particular, TNF-α and IL-6 stimulate the compensatory growth of hepatocytes through Nf-κB, mTOR and STAT3 in response to mitochondrial-induced apoptosis and, simultaneously, sustain a pro-survival tumor microenvironment via the paracrine/autocrine release of chemokines (CCL2, CCL7 and CXCL13) and cytokines (IL-1β, IL-6, TNF–α) [116,133,134,135,136].

The progression from NASH to HCC in obese people is even mediated by the unbalance between leptin and adiponectin. Leptin is involved in the inflammatory and fibrotic response via triggering the JAK/STAT, PI3KAkt and ERK signaling pathways [137]. Increased serum leptin was found in HCC patients with or without cirrhosis [138]. On the contrary, adiponectin can suppress tumor growth by either activating JNK-mediated mitochondrial apoptosis and caspases, or inhibiting Akt and STAT3 [137]. Nevertheless, low levels of adiponectin are unable to repress the KCs-mediated inflammatory response and promote HCC development [139].

## 5. Metabolic Reprogramming and Mitochondrial Dysfunction in HCC

Metabolic reprogramming is one of the key events determining the shifting from NASH to HCC, and a growing body of evidence has recognized it as a hallmark of hepatic cancer and not as an epiphenomenon of malignant transformation. Inflammation, fibrosis, ROS, fat accumulation, hypoxia and apoptotic signals challenge the hepatocytes to rewire liver metabolism in terms of cell survival, proliferation and, eventually, invasiveness. To support the high energy demand and macromolecule biosynthesis (lipids, nuclei acids, proteins), and to escape from disadvantageous conditions that may lead to programmed cell death, transformed hepatocytes metabolize glucose through glycolysis to produce ATP in the presence of oxygen, rather than proceeding with the mitochondrial respiratory chain. This rapid but low-efficiency phenomenon is paradoxically known as aerobic glycolysis or the Warburg effect, discovered by Otto Warburg in 1950 [9,11], and it allows the quick introduction and processing of glucose by accelerating glucose transport into the hepatocytes, increasing the kinetics of glycolytic enzymes and secreting glycolytic by-products, such as lactic acid.

Many studies have revealed the prognostic value of glucose transporters’ (GLUTs) upregulation in human HCCs. Among them, it has recently been suggested by Gao et al. that GLUT3 overexpression correlates with elevated α-fetoprotein (AFP) levels, tumor size, poor histological differentiation, and tumor node metastasis (TNM) stages, and it may predict the survival of HCC patients [140]. Hexokinase (HK), which traps glucose inside hepatocytes by its phosphorylation at the six-carbon (glucose-6 phosphate), further increases the glycolytic rate and, as in GLUTs, high HK activity sensitizes HCC cells to invasiveness and metastasis [141,142]. A microarray analysis of 153 HCC subjects revealed that HK2 was overexpressed in both dysplastic and carcinogenic tissues. Consistently, liver-specific HK2 knockout (KO) mice showed a reduced incidence of DEN-induced HCC, an ameliorated oxygen consumption rate (OCR), and a response to Sorafenib, a multikinase inhibitor exploited as a pharmacological approach in advanced HCCs [141]. Notably, a novel HK domain containing 1 (HKDC1) isoform led to liver tumorigenesis via the Wnt/β-catenin cascade, and was associated with poor outcome and metastasis in HCC [143]. Glyceraldehyde-3-phosphate dehydrogenase (GAPDH), catalyzing the sixth step of glycolysis, exerts pleiotropic roles, and is stably expressed within cells to the extent that it is duly used as a reference gene in research fields. However, it has been demonstrated that GAPDH may regulate mTOR-C1 signaling and cell growth according to glucose availability [144]. In addition, GAPDH can interact with the voltage-dependent anion channel (VDAC1) to activate apoptosis by inducing IMMs permeabilization, the loss of inner transmembrane potential, matrix swelling, the release of cytochrome c, and AIF [145]. In GAPDH transgenic mice and in HCC murine models induced by DEN, GAPDH overexpression accelerated tumor development and progression by regulating inflammatory cytokines (Il-6, Il-1β, Mcp1, Icam1, Vcam1) at the transcriptional level and redirecting metabolic intermediates of glycolytic flux towards the one-carbon cycle, which is essential for cell proliferation [146]. Higher mRNA levels of GAPDH have also been observed in HCC biopsies compared to non-HCC adjacent sides and normal liver [147].

According to Warburg’s theory, the aerobic glycolysis adopted by tumoral cells results from changes in the mitochondrial metabolism and, specifically, it involves pyruvate fate. At the final phases of glycolysis, pyruvate kinase (PK) converts phosphoenolpyruvate (PEP) into pyruvate, which, in turn, can be used to generate acetyl-CoA by pyruvate dehydrogenase (PDH), thus linking cytosolic glycolysis to mitochondrial TCA. In HCCs, hypoxic conditions stimulate HIF-1α to inhibit PDH, and pyruvic acid is re-routed towards lactate production through lactate dehydrogenase (LDH). Several findings have revealed that HFD models alter hepatic GLUT1/3 expression and aggravate LDH levels, supporting the pathogenic role of lipids in mitochondrial dysfunction and glucidic reprogramming from the early stages of NAFLD [148]. Moreover, lactate overflowing may contribute to H_2_O_2_ production in isolated mitochondria from murine hepatic tissue, directly taking part in oxidative damage [149]. A meta-analysis including 10 non-randomized controlled studies highlighted that a pre-operative increase in LDH levels was significantly associated with poor life-expectancy in individuals affected by HCC, thus appearing as a promising factor to evaluate HCC prognosis [150].

Finally, the abundance of mitochondrial pyruvate carriers (MPC) that transfer pyruvic acid from cytosol to mitochondria for oxidation is reduced in HCCs, thereby sustaining tumorigenesis and glucose utilization. It was observed that low MPC1 protein expression may represent an attractive biomarker for monitoring tumor relapse over the time period following hepatectomy in HCC patients [151]. All this notwithstanding, Tompkins et al. showed that MPC disruption in a murine model of HCC induced by N-nitrosodiethylamine plus carbon tetrachloride (CCL_4_) administration impairs hepatocarcinogenesis by hijacking and redirecting glutamine onto the Krebs cycle, rather than glutathione synthesis, the downregulation of which impedes tumor growth [152].

### 5.1. Mitochondrial Dynamics in HCC: Embarking on New Paths for Novel Therapeutic Targets

Contradictory results concerning the alterations in mitobiogenesis and mitophagy have led to misleading information about the role of mitochondria in NAFLD-related HCC, and need to be deeply elucidated. Alterations in mitochondrial morphology towards a fragmented shape are suggestive of carcinogenesis and a metastatic phenotype. Consistently, low levels of fusion proteins have been observed in human HCC micrographs compared to their non-cancerous counterparts, and were associated with cancer development, progression and refractoriness to medical treatments. MFN1 reduction was correlated either with EMT and the reprogramming of glucose metabolism alongside vascular invasion and poor outcome in HCC patients [77], while MFN2 overexpression induced cell death by enhancing cytochrome c release, Ca^2+^ entry and lowering mitochondrial membrane potential in HepG2 cells, thus inhibiting HCC cell growth [153,154]. Up to 40% of HCC patients showed decreased hepatic expression of OPA1 isoforms [155,156]. Among them, OPA1-Exon4b is mandatory for maintenance of mitochondrial respiration and IMMs potential, and it also regulates mitochondrial bioenergetics at the transcriptional level by binding the D-loop region of mtDNA [156]. In HCC, the inhibition of OPA1-Exon4b causes low ATP synthesis and compromises membrane potential along with alterations of TFAM distribution, an essential factor in determining the abundance of the mitochondrial genome by regulating its packaging, stability, and replication [156]. The low mtDNA copy number in HCC has been associated with reduced proliferation and migration, although it improved chemotherapy sensitivity [157].

In contrast to aforementioned evidence, Li et al. revealed that *Mfn1* and *Opa1* knockdown hampered cell proliferation paralleled by apoptosis, low O_2_ consumption and ATP production in HCC organoids and in vivo models [158]. Furthermore, the mitochondrial dimension was found to be larger in 10 HCC specimens compared to non-tumoral tissues, suggesting that mitochondrial elongation may sustain cancer metabolism and growth [158]. These findings were supported by analyzing five HCC cohorts from the Oncomine microarray database (https://www.oncomine.org) (accessed on 4 January 2020), including more than 400 HCC patients for whom RNA-seq data were available [158]. It has been reported that OPA1 expression is strictly involved in sensitizing HCC cells to cytotoxicity induced by Sorafenib [159]. Zhao and collaborators demonstrated that Sorafenib exposure led to mitochondrial fragmentation by downregulating OPA1 and enhancing apoptosis through cardiolipin peroxidation. Additionally, Sorafenib mixed with FH535, a β-catenin antagonist, synergically targeted complexes of respiratory chain and reduced aerobic glycolysis, suggesting that Sorafenib-FH535 treatment may overcome the low efficacy of current single pharmacotherapies used in HCC [160].

Emerging evidence has pointed out that mitochondrial fission promoted proliferation, tumor microenvironment and invasiveness. In 15 HCC tissues, Huang et al. observed a prevalence of globular mitochondria with a lower length compared to those of matched non-HCC tissues accompanied by high DRP1 protein expression and reduced mitofusins. Fragmented mitochondria staunching in the hepatocytes exacerbate ROS production, which stimulates Akt-mediated NF-kB activation and cell cycle activity, alongside the inhibition of the *TP53* gene and autophagy [131]. DRP1 also enhances HCC growth by promoting G1/S phase transition through coordinately modulating NF-kB and p53 pathways. Consistently, *Drp1* silencing led to cell cycle arrest in both in vitro and preclinical models [23]. In HCC mice, Drp1 overexpression caused mtDNA stress, promoting Ccl2 secretion and the infiltration of tumor-associated macrophages (TAMs). Moreover, DRP1 levels correlated with the percentage of TAMs in 69 HCC biopsies [161], and modulate the efficacy of chemotherapy response. Cisplatin coupled to DRP1 inhibitor (Mdivi-1) synergically activated apoptosis by augmenting the Bcl2-associated X/Bcl extra-large (Bax/Bcl-xL) ratio and increasing both the mitochondrial membrane permeability and cytochrome c release.

### 5.2. Recovery of Mitophagy in HCC: Friend or Foe?

There is mounting evidence highlighting that mitochondrial quality control and mitophagy may attenuate chronic liver injury in the NAFLD/NASH background, and consequently HCC occurrence, by rescuing mitochondrial integrity and functions [19,72,84,162,163,164]. As previously described, deranged mitochondria increase oxidative damage and the release of hepatocyte-derived mito-DAMPs (i.e., mtDNA) which may recruit Kupffer cells, exacerbate innate immune response, and activate HSCs, thus promoting carcinogenesis [98]. Empowering mitophagic processes by overexpressing E3 ubiquitin ligases (*Fundc1*, *Bnip3*) prevents DEN-induced HCC in mice and alleviates inflammasome cascade, JNK signaling, cell proliferation, migration, and invasiveness [165,166]. Excess mitophagy may cause ATP shortage and Ca^2+^ mobilization from ER, affecting filamentous actin polymerization and the lamellipodium-based migration of malignant hepatocytes [166]. Consistently, *Parkin* null mice showed reduced body weight but also showed liver enlargement as they spontaneously developed advanced HCC paralleled with high AFP and β-catenin expression, thus recapitulating human HCC [167]. Parkin downregulation was detected in up to 80% of HCC cases and PINK1 inhibition was associated with poor clinical outcome [168,169,170]. Notably, hypoxia may reduce PINK1/Parkin-dependent mitophagy, resulting in the loss of mitochondrial cristae, mass, and augmented CSC progenitors (Figure 2) [168].

All this notwithstanding, unbalanced mitophagy may initiate and/or accelerate hepatocarcinogenesis to the extent that blocking mitophagy may restore Sorafenib sensitivity [171]. Huang et al. showed that the number of mitochondria in the fission state was frequently high in HCC tissues compared to adjacent non-tumor ones, and these morphological alterations were accompanied by enhanced autophagic processes [131]. Furthermore, ERK/HIF-1α-mediated BNIP3 upregulation promoted the acquisition of anoikis resistance, a type of programmed cell death occurring when cells lose their attachment to the ECM and to neighboring cells [172]. Liu and coworkers demonstrated that the activation of PINK1-dependent mitophagy both provides a selective removal of the onco-suppressor p53 and maintains hepatic stem cell population [173].

### 5.3. Metabolic and Epigenetic Dysregulation of Mitochondrial Metabolism in HCC: A Huge Variability

The regulation of mitochondrial turnover mostly occurs in response to low-energy conditions and through AMPK/SIRT/PGC1α nutrient sensors. Controversial pre-clinical and human studies reported that both up- and downregulation of the AMPK/SIRTs/PGC1α network can lead to glycolytic reprogramming and HCC [174], thereby making it mandatory to focus research efforts to elucidate discrepancies.

Several investigations reported that PGC1α deficiency was associated with the degeneration of mitochondrial morphology [175], the de-differentiation of cultured hepatocytes [175,176], and a high glycolytic rate in human HCCs [177]. Downregulation of PGC1α expression has been reported in publicly available repositories, such as the Cancer Genome Atlas (TCGA, https://www.cbioportal.org/) (accessed on 4 January 2017) and GSE14520 (https://www.ncbi.nlm.nih.gov/geo/info/overview.html) (accessed on 2 January 2021), containing transcriptomic analyses of HCC patients, and in clinical findings [175,178]. Recently, the loss of PGC1α was correlated with poor prognosis and favored the Warburg effect through the WNT/β-catenin/pyruvate dehydrogenase kinase (PDK) axis, and also metastasizing in HCC patients [178,179]. Likewise, low SIRT3 expression was associated with serum AFP levels, which has both diagnostic and prognostic value in the context of HCC, tumor multiplicity and high relapse rate, thus representing a prognostic marker of overall survival in HCC patients [66]. Importantly, it has been reported that SIRT3 can link to four hallmarks of cancer, such as genomic instability, sustained proliferation, dysregulated energetic status, and tumor-promoting inflammation. However, in HCCs, SIRT3 acts as a tumor suppressor, as it mitigates ROS-induced hepatocellular injury and interacts with glycogen synthase kinase 3 beta (GSK-3β) to induce Bax translocation to the mitochondria, causing apoptosis [67].

On the other hand, it has been reported by Bhalla et al. that *Pgc1α*^−/−^ mice were protected against DEN-induced liver cancer, and Pgc1α overexpression in mice induced gene expression reprogramming, supporting DNL, glycolysis, and oxidative metabolism [16]. In keeping with these findings, several studies showed that sirtuins, NAD^+^-dependent epigenetic modifiers of liver metabolism, mitochondrial functionality, telomere length and genomic stability, were repeatedly found to be overexpressed in human HCC cell lines such as HKC1-4, SNU-423, HKC1-2, PLC5 SNU-449, SK-Hep-1, Huh-7, HepG2 and Hep3B, and in multiple malignancies [180,181,182,183,184]. SIRT1 upregulation triggers mitobiogenesis via PGC1α and correlates with tumor microvascular invasion, advanced TNM score and predicted HCC recurrence [184]. *Sirt1* genetic ablation (*Sirt1*^−/−^) mitigated tumor growth and invasiveness in both in vitro and in vivo models. Interestingly, when mitochondrial biogenesis was re-activated by overexpressing Pgc1α in *Sirt1*^−/−^ mice, they re-developed an aggressive HCC phenotype, suggesting that mitobiogenesis may sustain tumor progression [184]. It has been shown that SIRT6 overexpression favors hepatoma cell proliferation through the extracellular signal-regulated kinases 1/2 (ERK1/2) pathway [183,185], whereas SIRT2 plays a critical role in promoting HCC metastasis and invasion rather than cell growth by re-routing liver cancer metabolism [182]. SIRT2 mediated mitochondrial deacetylation and the stabilization of phosphoenolpyruvate carboxykinase (PEPCK), involved in gluconeogenesis, by catalyzing the conversion of oxaloacetate into PEP, and glutaminase (GLS), which converts glutamine into glutamate, thus replenishing the Krebs cycle of glucidic and aminoacidic substrates [182]. Either PGC1α-mediated mitochondrial biogenesis or the fueled citric acid cycle enhanced NADH/ATP production and O_2_ consumption rate, and both contribute to the occurrence of HCC metastasis [182,186]. In addition, both SIRT2 and SIRT6 inhibit the adhesion properties of E-cadherin proteins, further supporting their role in cell migration and invasiveness [182,183].

Just recently, Zhao and collaborators provided the first evidence that the long non-coding RNA (lncRNA) MALAT1 controls metabolic reprogramming in hepatocytes, and its downregulation increased the number of swollen mitochondria, along with dampening OXPHOS, ATP production and mtDNA copy number. Additionally, *MALAT1*-deficient cells affected mitophagy by reducing PINK1, SQSTM1/p62, NDP52, BNIP3 and LC3 expression, thereby supporting its oncogenic role as an lncRNA in HCC onset [187].

### 5.4. The Impact of Hypoxia on Hepatic Metabolic Reprogramming and Mitodynamisms

The liver is the major organ that stocks nutritional surplus into glycogen, LDs, cholesterols, and supplies energy-producing substrates to the peripheral tissues even under fasting conditions. A remarkable amount of O_2_ is required to regulate anabolic and catabolic processes occurring in the hepatocytes, thus causing a steep O_2_ gradient throughout the hepatic lobules that may get back to normoxia through cell-adapting systems [119]. HIF-1 and HIF-2, oxygen-sensitive transcription factors recognizing hypoxia response elements (HRE) on promoter regions, mediate cellular adaptation to low oxygen and regulate both glucose and lipid metabolism, respectively [188]. Excessive dietary intake together with fatty liver, necroinflammation, fibrotic scars and tissue regeneration may bother hepatic oxygen distribution, thus precipitating pathological hypoxia. It has been reported that HFD provoked hepatic hypoxia and impaired mitochondrial dynamics and functions (i.e., defective β-oxidation) through the HIF-2/PPARα pathway, thereby exacerbating NAFLD progression [188,189,190]. Fat-laden hepatocytes receiving cobalt chloride (CoCl2) to mimic hypoxia increased mitochondrial superoxide production and released extracellular vesicles enriched in chemoattractant cytokines (i.e., IL-1β, IL-6, TNF-α, iNOS, NLRP3) for KCs and inflammasome activation [168,191]. DEN-treated mice fed with a western diet showed increased expression of both HIF-1α and pro-inflammatory cytokines, allowing the recruitment of TAM with a M2 phenotype and the switching from NASH to HCC [118].

Under the hypoxic environment, HIF-2 overexpression may drive NAFLD–HCC development by triggering the PI3K/Akt/mTOR cascade and inducing lipid reprogramming [192], while HIF-1α stabilization forces the switching from OXPHOS to aerobic glycolysis by upregulating glycolytic enzymes and LDH [193]. Moreover, low oxygen availability induced high mobility group box 1 (HMGB1)-TLR9 binding, which mediates PGC1α phosphorylation and activation, thereby sustaining mitochondrial biogenesis and cell proliferation in in vitro, in vivo and human HCC samples [194]. It has been shown that hypoxia causes inefficient electron transfer to the mitochondrial respiratory chain, and affects mitophagy. Chiu and collaborators found an HRE consensus sequence on the promoter of the Hairy/enhancer-of-split related with YRPW motif protein 1 (HEY1) gene, belonging to NOTCH signaling. HEY1 was overexpressed in HCC patients who underwent surgical resection and in 49 HCC cases from the TCGA dataset. HEY1 transcriptionally represses PINK1 in Huh7, reduces mitochondrial mass and alters inner cristae morphology [168].

## 6. The Link among NAFLD, Mitochondrial Dysfunction and HCC: The Relevance of Genetics

Environmental factors, above all IR and obesity, influence NAFLD pathogenesis. However, it has emerged that there is a substantial variability in hepatic lipid deposition among individuals with the same grade of adiposity, raising the possibility that several other risk factors may participate in steatosis development. Familial, twin, and epidemiological studies indicated that NAFLD has a strong heritable component, which contributes to the huge inter-individual phenotypic variability. Dongiovanni et al. demonstrated that hepatic fat accumulation represents the main driver of the progression to the end-stage of liver damage in genetically predisposed individuals, and recently proposed a detailed review including all the candidate genes related to NAFLD susceptibility [195,196].

Currently, the rs738409 C >G single nucleotide polymorphism (SNP) in the *Patatin-like phospholipase domain containing 3* gene (*PNPLA3* or adiponutrin) is the major genetic variant associated with NAFLD onset and its progressive forms, including HCC. PNPLA3 is mainly localized on the ER and LDs surface in hepatocytes, adipocytes and HSCs, and it may be transcriptionally induced or post-translationally modified to provide TG hydrolysis during the post-prandial or hyper-insulinemic state. Patients carrying the G allele lost PNPLA3 enzymatic activity, which impedes TG disposal and interferes with the activity of other lipases, such as PNPLA2 [196,197]. Beyond the triacylglycerol remodeling, PNPLA3 exerts widespread effects on human liver metabolome [198], influencing mitochondrial functions, glucose reprogramming and tumorigenesis. Huh-7 hepatoma cells overexpressing the PNPLA3 I148M variant showed high levels of lactate and γ-glutamyl-amino acids, thus mirroring the metabolic switching to aerobic glycolysis and mitochondrial failure, respectively [198]. Moreover, the rs738409 SNP impacts retinol secretion in HSC cells, leading to the myofibroblast-like phenotype and collagen deposition, and boosting fibrogenesis in NASH subjects [199]. In a small cohort of 54 NAFLD subjects, it has been demonstrated that carriers of the G risk allele had a severe profile of liver disease, characterized by enhanced steatosis, activation of pro-inflammatory pathways and an increased proliferative activity of hepatocytes [200]. Interestingly, Bruschi et al. demonstrated that the presence of I148M substitution in the *PNPLA3* gene further affected metabolic reprogramming in TGFβ-activated HSCs, shifting towards aerobic glycolysis, lactate release and the activation of YAP/Hedgehog signaling [201].

The rs641738 C > T variant in the *Membrane bound o-acyltransferase domain-containing 7*/*Transmembrane channel-like 4* (*MBOAT7*/*TMC4*) locus, encoding the MBOAT7 enzyme, was associated with the entire spectrum of NAFLD, including HCC [202]. Recently, the role of the *MBOAT7* variant in NAFLD progression has been evaluated in a large meta-analysis. Data were collected from 1047.265 subjects, of whom 8303 had liver biopsies, and displayed a correlation between the T minor allele and hepatic fat deposition, ALT levels, and advanced stages of NAFLD, such as fibrosis and HCC. In particular, carriers of the rs641738 variant show a 30% risk of developing HCC compared to non-carriers [203]. Physiologically, MBOAT7 localizes on MAMs and mediates phosphatidylinositol (PI) acyl-chain remodeling in the Land’s cycle. Our group demonstrated that hepatic MBOAT7 expression is reduced during hyperinsulinemia and by the presence of the rs641738 C > T variant [204,205]. MBOAT7 downregulation induces an enrichment of saturated PIs, which are shunted towards the synthesis of TGs, thus contributing to fat accumulation. Though no evidence linking MBOAT7, mitochondrial lifecycle and metabolic reprogramming has been reported, it could be postulated that the wealth of saturated lipids induced by MBOAT7 downregulation may affect membrane composition and dynamics, possibly breaking ER–mitochondria communications.

The rs58542926 C > T variant in the *Transmembrane 6 Superfamily member 2* (TM6SF2) gene induces TM6SF2 loss-of-function and hastens its hepatic protein turnover [206]. TM6SF2 dwells on ER-Golgi compartments where fat biosynthesis, LDs and lipoprotein formation occur. TM6SF2 inactivation induced by the presence of the polymorphisms impairs the assembly and trafficking of very low-density lipoprotein (VLDL), which remains trapped in hepatocytes [206]. In Huh-7 cells, *TM6SF2* deficiency reduces the amount of PUFAs, along with causing alterations in mitochondrial β-oxidation and higher numbers of lysosomal compartments [207]. In the small intestine of zebrafish, the *TM6SF2* loss-of-function induces changes in ER architecture appearing with enlarged cisternae, supporting the notion that TM6SF2 may impact organelles’ morphology [208]. However, the rs58542926 polymorphism has been associated with the NAFLD/NASH spectrum, but its role in HCC development remains to be explored. A meta-analysis including 24,147 subjects affected by chronic liver disorders revealed that the presence of the T risk allele was correlated with a higher risk of developing NAFLD and its advanced stages, such as HCC [209]. Raksayot et al. performed a cross-sectional study in a cohort of 502 NAFLD patients and observed that carriers of the T allele are at a higher risk of HCC progression [210].

To delve inside the mechanisms underlying NAFLD pathogenesis and to investigate possible synergisms among *PNPLA3*, *MBOAT7* and *TM6SF2* leading to hepatocytic metabolic rewiring, our group has generated in vitro models of genetic NAFLD. We stably silenced *MBOAT7* (MBOAT7^−/−^), *TM6SF2* (TM6SF2^−/−^), or both genes (MBOAT7^−/−^TM6SF2^−/−^), in HepG2 cells, homozygous for the I48M *PNPLA3* variant, by exploiting clustered regularly interspaced short palindromic repeats/CRISPR-associated protein 9 (CRISPR/Cas9) technology [10,11,205]. MBOAT7^−/−^ spontaneously accumulated giant LDs associated with a dramatic increment in ROS and peroxides levels, while TM6SF2^−/−^ and MBOAT7^−/−^TM6SF2^−/−^ models showed mitochondria with small and globular shapes, the loss of cisterns’ architecture, and ultrastructural electron density, suggestive of mitochondria degeneration. The numbers of mitochondria were progressively increased in all mutated cell lines, suggesting that either *MBOAT7* and/or TM6SF2 deficiency impact mitochondrial biogenesis. Notably, the compound knockout re-routed its metabolism towards glucose-dependent ATP production, enhancing glycolytic enzymes, LDH and lactate release, thereby suggesting that the depletion of both *MBOAT7*/*TM6SF2* combined with the genetic background of I148M *PNPLA3* may affect mitochondria turnover, possibly accelerating metabolic reprogramming [10,13].

Recently, the opportunity has emerged to translate the genetics into clinics by aggregating these genetic variants into polygenic risk scores, which may better discriminate NAFLD patients who are at-risk of developing progressive liver damage and HCC [211]. In a large cohort of biopsied NAFLD patients, it has been observed that the cumulative number of risk alleles is associated with serum markers of disease severity and increased risk of developing HCC [10,212,213]. A cross-sectional study consisting of 2566 NAFLD participants evaluated the impact of these genetic polymorphisms on hepatocytic fat accumulation in HCC progression. For this purpose, the authors generated a polygenic risk score based on the presence of variants in *PNPLA3*, *TM6SF2 MBOAT7* and *GCKR* genes, which was able to predict HCC occurrence much more effectively than the presence of a single genetic variant [211].

### 6.1. Mitochondrial Polymorphisms Are Correlated with NAFLD Pathogenesis

Several polymorphisms in mitochondrial genes have been associated with NAFLD development and progression. In genetically modified mice, the non-synonymous nt7778 G > T genetic variation in the mitochondrial ATP synthase protein 8 (mt-ATP8) increased susceptibility to diet-induced NASH [214]. The identification of mitochondrial haplotypes was even associated with NAFLD predisposition, opening new avenues for mito-genetic screening in patients, and new experimental applications [212].

Manganese-dependent SOD, encoded by the nuclear *SOD2* gene, mitigates oxidative damage by catalyzing the conversion of superoxide radicals to hydrogen peroxide. The rs4880 C47T variant in the *SOD2* gene encodes the valine to alanine amino acid substitution at position 16 in the signal region targeting the protein to the mitochondrial matrix. The C47T mutation causes a reduction in MnSOD2 activity and the consequent failure to neutralize superoxide radicals. In case–control and familial studies, Al-Serri et al. demonstrated that the inherited T risk allele was an independent predictor of NASH severity and was strictly associated with fibrosis in both adults and children [213]. Conversely, the −866 G > A polymorphism localized in the promoter region of the *UCP2* gene, involved in heat dissipation, has been associated with a reduced risk of obesity [215]. The A allele promotes UCP2 overexpression in the liver and has a protective role in progression from simple steatosis to NASH [216]. Likewise, the rs1800849 −55 C/T *UCP3* variant ameliorates the circulating lipid profile and correlates with loss of body weight [217]. These findings were not confirmed by Aller et al. and Qian et al., who associated both the −866 G > A polymorphism and the rs1800849 variant with higher risk of IR, obesity, lower levels of adiponectin, severe steatosis, and inflammation in NAFLD subjects [218,219].

Polymorphisms in sirtuins further contribute to the regulation of mitochondrial functionality and dynamics, possibly contributing to NAFLD/NASH advancement and its cardiovascular comorbidities [220]. Patients carrying the rs11246020 variant (V208I) in the *SIRT3* gene displayed a higher susceptibility to NAFLD. Consistently, *Sirt3* knockout mice fed an HFD showed IR and a worsened adiposity and NASH [221]. The rs107251 in the *SIRT6* gene affected SIRT6 activity, influencing its role in DNA repair and the maintenance of telomeric chromatin [222]. It has been described that the rs7895833 G > A in the *SIRT1* gene represents a risk factor for body fat content and high diastolic blood pressure [222,223]. Interestingly, low SIRT1 levels were detected in 70 cirrhotic HCC patients carrying the rs7895833 variant, and SIRT1 reduction was inversely correlated with high AFP, Child–Pugh score and tumor stage [224].

Recently, rs2642438 A165T polymorphisms at the N-terminal domain of the *Mitochondrial amidoxime reducing component 1* (*MARC1*) gene have been described, localizing on MOMs. The A165T variant has been associated with low fat content in the liver and a reduced risk of NALD progression toward cirrhosis. Such findings were independently validated by Lukkonen et al., showing that carriers of the rs2642438 variant had alleviated NASH severity accompanied by an improvement of the hepatic lipid profile, mainly consisting of polyunsaturated phosphatidylcholines, thus suggesting that *MARC1* could represent a candidate therapeutic target [225,226].

### 6.2. Rare NAFLD Pathogenic Variants Are Involved in Switching towards HCC

Part of the missing hereditability in NAFLD may be attributed to rare genetic variants with a large effect size.

Rare mutations in the *telomerase reverse transcriptase* (*TERT*) promoter may arise in NAFLD–cirrhosis, in 10–20% of both low-grade and high-grade dysplastic nodules and in familial HCC, suggesting that *TERT* germline genetic variants may be involved in tumor initiation [227]. In a cohort of 40 NAFLD–HCC, 45 cirrhotic patients and 64 healthy controls, telomere length decreased with the progression of NAFLD towards cirrhosis and mainly with HCC [228]. Four rare mutations have emerged in the *hTERT* gene in NAFLD–HCC subjects, such as the Glu113Arg_fs*79 frameshift in the second exon, and three missense mutations (Ala67Val, Pro193Leu, Glu668Asp), which correlated with shorter telomere length. In particular, the Ala67Val and Glu668Asp SNPs led to TERT’s loss-of-function and decreased its hepatic expression. On the contrary, Pro193Leu substitution did not affect TERT catalytic activity but reduced its chromatin binding capacity [229]. Furthermore, in a cross-sectional study, it was observed that NAFLD–HCC patients showed an enrichment of rare genetic variants in *Regulator of telomere elongation helicase 1* (*RTEL1*) and *Telomeric repeat binding factor 2* (*TERF2*) genes, which are involved in telomere preservation, and in *RB1*, which mediates the oxidative stress response. Mutations in *STK11*, *TSC1*, *TSC2*, *NF2* and *SMAD4* candidate genes, which regulate cell growth and proliferation, were also strongly correlated to HCC risk [230].

HCC surveillance may be addressed to NAFLD subjects with a family history of hypobetalipoproteinaemia caused by *ApoB* mutations. The *ApoB* gene is involved in hepatic lipid metabolism, and its genetic variants lead to an impaired synthesis of ApoB100, with the consequent alteration of the hepatic VLDL export. Uncommon variants in the *ApoB* gene result in the impairment of VLDL export and the development of severe hepatic steatosis. Some of the genetic variants causative of ineffective ApoB100 synthesis may even alter the ApoB48 isoform expressed in the enterocytes, provoking the malabsorption of fat and insoluble vitamins, the retention of chylomicrons, and alterations of the intestinal barrier [231].

Finally, a novel association between variants in Sequestosome 1 (*SQSMT1*) and HCC onset has been identified in NAFLD–HCC patients. *SQSMT1* encodes the ubiquitin-binding protein p62, an autophagosome cargo protein that targets other proteins for selective autophagy. p62 contributes to Mallory–Denk bodies (MDBs), a cytoplasmatic protein aggregate found in several chronic liver diseases including NAFLD, as well as in HCC, and it is involved in the hepatocyte’s transformation through the activation of the mTOR pathway and the regulation of telomere length machinery [27,232].

## 7. Targeting Mitochondria in NAFLD–HCC: New Challenges for a Bright Future

The staging and treatment selection of HCC patients follow the Barcelona Clinic for Liver Cancer (BCLC) criteria employed by both the American and European Associations for the Study of Liver Diseases [120,233]. The BCLC system includes the evaluation of liver function, patient’s well-being and ability to perform daily activities, combined with the assessment of tumor number, mass, and spreading. Resection, LT and ablation may be curative for small tumors at early stages of BCLC classification (0, A), while subjects with intermediate BCLC score (B), presenting multifocal tumors without vascular invasion, usually undergo transarterial chemoembolization (TACE) as the first-line therapy. Interestingly, in NAFLD patients who developed HCC without cirrhosis, lifestyle changes and weight loss are encouraged to ameliorate steatosis, mitochondrial biogenesis and mitochondrial morphology, and to promote an anticancer immune microenvironment. However, the identification of NAFLD–HCC patients staging BCLC-0, BCLC-A and BCLC-B is challenging, and the majority of NAFLD–HCC cases are diagnosed at advanced stages (BCLC-C/D). To date, sorafenib is the only pharmacological therapy with proven benefits for HCC management, although it is poorly tolerated and increases life expectancy by 9–11 months [120]. Additionally, sorafenib proved to be ineffective in patients in the BCLC-D category, to which only palliative care is offered as the medical option.

Ongoing efforts strive towards early detection, preventive strategies, and the introduction of novel therapeutic approaches based on immunological modulation and/or surveillance, pan-tyrosine kinase inhibitors, and vaccination [120]. Moreover, several agents (i.e., vitamin E, vitamin B6 plus Coenzyme Q10), which indirectly improve mitochondrial activities and dynamics, have shown positive outcomes in NALFD management, and they are currently being tested for HCC medical applications. Vitamin E, the best antioxidant against mitochondrial damage, is considered the first-line treatment for non-cirrhotic and non-diabetic subjects affected by NAFLD/NASH [234]. Fantappiè and collaborators demonstrated that vitamin E prevents HCC development in mice by downregulating nitric oxide synthase (iNOS) and NADPH oxidase [235], and it also protects against ROS-induced DNA damage in HCC cells [236]. In a randomized controlled trial (NCT01964001), the administration of vitamin B6 alone or supplemented with Coenzyme Q10 significantly increased antioxidant enzymes and reduced oxidative stress, along with inflammation, in HCC patients after tumor resection [237,238].

Antihyperglycemic drugs, such as pioglitazone, a potent ligand of PPARγ, delay liver fibrosis and hepatocarcinogenesis in dietary NASH models and DEN-treated mice by enhancing AMPK pathways and mitobiogenesis [239]. Clinical evidence has suggested considering pioglitazone administration alone or preferably in combination with statins to prevent cirrhosis, LT and HCC occurrence in NAFLD/NASH subjects at high risk of cardiovascular complications [240]. Interestingly, a phase II clinical trial is currently evaluating the effect of rosiglitazone combined with nivolumab on immune response and mitochondrial activities in different solid tumors, including HCC (NCT04114136).

AMP mimics, such as aminoimidazole-4-carboxamide ribonucleoside (AICAR) and indirect AMPK activators (metformin), have provided advantageous effects for the management of metabolic disorders. It has been demonstrated that AICAR exposure suppresses aerobic glycolysis, reduces TCA intermediates and lactate production, and also dampens HSCs activation by inhibiting mTORC1 signaling [241,242]. Metformin blocks HIF1α-phosphofructokinase (PFK), which catalyzes the third step of glycolysis, and lactate release in hepatoma cells and tumor tissues, thereby attenuating HCC growth [243].

In recent years, the development of mitochondrial-based strategies has offered beneficial effects in metabolic diseases [11], to the extent that they may represent an interesting avenue to pursue for NAFLD-related HCC treatment, either as prophylaxis or patient care. Wang’s research group recently introduced the tri-phenyl-phosphine (TPP) groups into the copper–terpyridine complex, thus generating a novel molecule, called CTB, which exhibits mitochondrial-targeting properties [244]. CTB administration in HepG2 cells inhibits aerobic glycolysis and promotes DRP1 recruiting and the opening of mitochondrial pores, thus contributing to the activation of fission and mitophagy [244]. Furthermore, CTB affects TERT methylation and expression, leading to the inhibition of both cell senescence and the growth of implanted tumors in mice [245]. Parvalbumin, a mitochondrial Ca^2+^-buffering protein, dramatically lowered ROS production and invasiveness, and suppressed EMT in HCC cells by modulating the Ca^2+^ flow into mitochondria and rescuing the NAD^+^/SIRT3/SOD2 axis [246,247].

## 8. Concluding Remarks

Over the last two decades, NAFLD has shown an upsloping trend, mirroring both lifestyle changes (wealth in food supplies, lack of physical activity, etc.) and the increasing incidence of MetS worldwide. Up to now, NAFLD has represented the most common chronic liver disorder affecting both adult and pediatric populations, and it will rapidly become the foremost cause of LT and HCC due to the high efficacy of anti-viral therapies.

**Figure 3:**
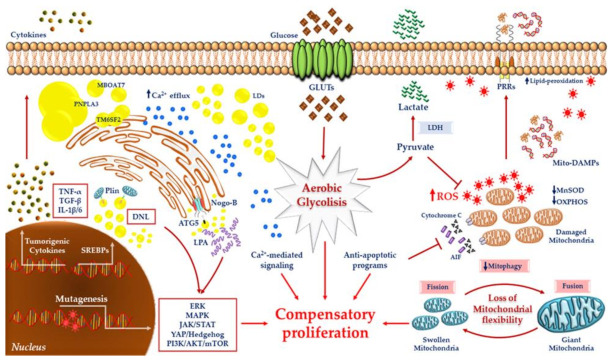
Mitochondrial pathways implicated in the pathogenesis of NASH-related HCC. Sedentary lifestyle, dietary habits and genetic background contribute to NAFLD development and its progression to NASH-driven HCC. The excess of hepatic fat accumulation promotes DNL, mitochondrial dysfunctions and the disruption of ER–mitochondrial contact sites. Consequently, the increased content of LDs may favor either lipotoxicity or energetic substrates for cell viability. The loss of mitochondrial flexibility and reduced mitophagy exacerbate the number of degenerated mitochondria, which produce ROS and release cytochrome C and AIF, thus activating apoptosis. The oxidative stress overwhelms antioxidant defenses, affects OXPHOS capacity, and triggers inflammatory signals (tumorigenic cytokines). In addition, ROS induce the mutagenesis of both nuclear DNA and mtDNA, causing the aberrant activation of proliferative pathways and the delivery of mito-DAMPs, respectively. Mito-Damps, including mitochondrial formyl-peptides and mtDNA fragments, could activate PRR receptors on the hepatocellular surface, KCs and HSCs, prompting inflammation and fibrosis. ROS-induced pro-survival signaling (i.e., JAK/STAT, ERK, MAPK) is able to counteract cell death by inducing the degradation of onco-suppressors and expanding the amount of hepatic progenitor cells. Moreover, the interruption of ER–mitochondria communication raises the efflux of cytosolic Ca^2+^, further contributing to ROS production and mutagenesis. Finally, hepatocytes undergo metabolic reprogramming, characterized by enhanced glucose uptake, high rates of glycolysis, and lactate production, which is rapidly secreted to avoid cytosolic acidification. Overall, a lipid-rich microenviroment combined with early loss of mitochondrial adaptability, both hallmarks of NAFLD onset and progression, may rearrange hepatocellular metabolism and the interplay between hepatocytes and non-parenchymal cells in order to overcome an adverse environment and trigger tumorigenesis.

One of the key steps of NAFLD pathophysiology is the burst of mitochondrial dysfunction, which may arise from the earliest events of fatty liver in response to environmental, genetic and epigenetic factors, supporting a new view of NAFLD as a mitochondrial disorder. Ever-growing findings have indicated that mitochondria adapt in number, size, morphology, and organelle communications to overcome energy demands and to provide essential macromolecules for cell viability. Throughout the NAFLD course, hepatocytes are exposed to endogenous hardship and extrinsic constrains, rendering burdensome the survival of parenchymal cells. In this context, mitochondria actively participate to evade apoptotis, to escape inflammation, to induce mutagenesis through oxidative damage, and to rewire hepatocellular metabolism with the goal of favoring the clonal selection of malignant hepatocytes forming tumor mass (Figure 3).

The study of mitochondrial dynamics in NAFLD/NASH and NAFLD-related HCC is challenging, and currently still in the early phase, due to the high phenotypic variability that characterizes this complex disease. Liver resection and transplantation represent the ongoing curative options in NASH-related HCC. Therefore, deep knowledge of the mitochondrial lifecycle and networking in metabolic disorders, especially those severely impacting HCC susceptibility, may offer novel insights for research purposes. On the other hand, it may pave the way for the development of new mitochondrial-based strategies, which could be introduced into clinics for either preemptive use or as accompaniments of approved treatments already used in HCC patient care.

## Data Availability

Not applicable.

## Abbreviations

CC: acetyl-CoA carboxylase; ACLY, ATP-citrate lyase; AFP, α-fetoprotein; AIF, apoptosis induced factor; AMLN; high trans-fat, high fructose and high-cholesterol; AMPK, 5′AMP-activated protein kinase; ATG5, autophagy related 5; ATP, adenosine Triphosphate; BCL, B-cell lymphoma; Bax/Bcl-xL, BCL2 associated X/Bcl-extra-large; BNIP3, BCL2/adenovirus E1B 19 kDa-protein-interacting protein 3; Ca^2+^, calcium; CCL, C-C-motif chemokine ligand; CDE; choline-deficient, ethionine-supplemented diet; CPT1/2, Carnitine Palmitoyl-Transferase 1/2; CRISPR/Cas9, clustered regularly interspaced short palindromic repeats/CRISPR-associated protein 9; CSC, cancer stem cells; DEN, diethylnitrosamine; DNL, de novo lipogenesis; DRP1, dynamin-related protein 1; EMT, epithelial–mesenchymal transition; ER, endoplasmic reticulum; ETC, electron transport chain; FADS, fatty acid desaturase; FASN; fatty acid synthase; FFAs, free fatty acids; Fis1, fission 1; FGF21, fibroblast growth factor 21; FUNDC1, FUN14 domain-containing 1; GAPDH, glyceraldehyde-3-phosphate dehydrogenase; GPX1, glutathione peroxidase 1; GLUTs, glucose transporters; GSSG/GSH, glutathione disulfide/glutathione; GST, glutathione transferase; H2O2, hydrogen peroxide; HBV, hepatitis B viral; HCC, hepatocellular carcinoma; HCV, hepatitis C viral; HFD, high-fat diet; HEY, YRPW motif protein 1; HK, hexokinase; HRE, hypoxia response elements; HSCs, hepatic stellate cells; IL-6, interleukin 6; IMMs, inner mitochondrial membranes; IR, insulin resistance; IRF1, interferon regulatory factor-dependent type 1; JNK, c-Jun NH2-terminal kinase; KCs, Kupffer cells; LC3, light chain 3; LDH, lactate dehydrogenase; LDL, low-density lipoproteins; LDs, lipid droplets; LIR, LC3-interactive region; LPA, lysophosphatidic acid mediator; LT, liver transplantation; LXR, liver X receptor; MAMs, mitochondrial-associated ER membranes; MAPKs, mitogen-activated protein kinases; MDVs, mitochondrial-derived vesicles; MetS, metabolic syndrome; Mff; mitochondrial fission factor; MFNs, Mitofusins; MiD49/50, mitochondrial dynamics 49/50; MnSOD, manganese superoxide dismutase; MOMs, mitochondrial outer membranes; MPC, mitochondrial pyruvate carriers; mtDNA, mitochondrial DNA; mTOR, mammalian target of rapamycin; MTP, mitochondrial trifunctional protein; MUFAs, mono-unsatured fatty acids; NAFLD, nonalcoholic fatty liver disease; NASH, nonalcoholic steatohepatitis; Nf-κB, nuclear factor-kappa-light-chain-enhancer of activated B cells; NLR, NOD-like receptors; NOD, nucleotide binding oligomerization domain; NRF1/2, nuclear respiratory factor 1/2; O_2_, oxygen; OA, oleic acid; Opa1, optic atrophy 1; OXPHOS, oxidative phosphorylation; PA, palmitic acid; PDH, pyruvate dehydrogenase; PDM, peri-droplets mitochondria; PEP, phosphoenolpyruvate; PGC1s, peroxisome proliferator-activated receptor-γ coactivator 1; PI3K, phosphoinositide 3-kinase; PINK1, PTEN-induced kinase 1; PK, pyruvate kinase PLIN, perilipin; PPAR, peroxisome proliferator-activated receptor; PRRs, pattern recognition receptors; PTEN, phosphatase and tensin homologue; PUFAs; polyunsaturated fatty acids; ROS, reactive oxygen species; SCD, stearoyl-CoA desaturase; SIRT, Sirtuin; SREBP1/2, sterol regulatory element-binding protein 1/2; STAT3, signal transducer and activator of transcription 3; T2DM, type 2 diabetes mellitus; TAMs, tumor-associated macrophages; TCA, tricarboxylic acid; TGs, triglycerides; CPT TLR, toll-like receptor; TNF-α, tumor necrosis factor α; TNM, tumor node metastasis; Tri-phenyl-phosphine, (TPP); TRAIL, TNF-related apoptosis inducing ligand; UCP2, uncoupling protein 2; UPRmt, mitochondrial unfolded protein response; VLDL, very-low density lipoproteins; YAP, yes-associated protein.
